# Biosynthetic gene cluster signature profiles of pathogenic Gram-negative bacteria isolated from Egyptian clinical settings

**DOI:** 10.1128/spectrum.01344-23

**Published:** 2023-09-14

**Authors:** Nehal Adel Abdelsalam, Mohamed Elhadidy, Nehal A. Saif, Salma W. Elsayed, Shaimaa F. Mouftah, Ahmed A. Sayed, Laila Ziko

**Affiliations:** 1 Biomedical Sciences Program, University of Science and Technology, Zewail City of Science and Technology, Giza, Egypt; 2 Center for Genomics, Helmy Institute for Medical Sciences, Zewail City of Science and Technology, Giza, Egypt; 3 Department of Microbiology and Immunology, Faculty of Pharmacy, Cairo University, Cairo, Egypt; 4 Department of Bacteriology, Mycology and Immunology, Faculty of Veterinary Medicine, Mansoura University, Mansoura, Egypt; 5 Department of Microbiology and Immunology, Faculty of Pharmacy, Ain Shams University, Cairo, Egypt; 6 Genomic research program, Basic research department, Children’s Cancer Hospital Egypt, Cairo, Egypt; 7 Department of Biochemistry, Faculty of Science, Ain Shams University, Cairo, Egypt; 8 School of Life and Medical Sciences, University of Hertfordshire, Hosted by Global Academic Foundation, Cairo, Egypt; Nevada State Public Health Laboratory, Reno, Nevada, USA

**Keywords:** ESKAPE pathogens, specialized metabolites, biosynthetic gene clusters, natural products

## Abstract

Biosynthetic gene clusters (BGCs) are a subset of consecutive genes present within a variety of organisms to produce specialized metabolites (SMs). These SMs are becoming a cornerstone to produce multiple medications including antibacterial and anticancer agents. Natural products (NPs) also play a pivotal role in enhancing the virulence of ESKAPE pathogens (*Enterococcus faecium*, *Staphylococcus aureus*, *Klebsiella pneumoniae*, *Acinetobacter baumannii*, *Pseudomonas aeruginosa*, and *Enterobacter* spp.), which represent a global health threat. We aimed to sequence and computationally analyze the BGCs present in 66 strains pertaining to three different ESKAPE pathogenic species: 21 *A. baumannii*, 28 *K. pneumoniae*, and 17 *P. aeruginosa* strains recovered from clinical settings in Egypt. DNA was extracted using QIAamp DNA Mini kit and Illumina NextSeq 550 was used for whole-genome sequencing. The sequences were quality-filtered by fastp and assembled by Unicycler. BGCs were detected by antiSMASH, BAGEL, GECCO, and PRISM, and aligned using Clinker. The highest abundance of BGCs was detected in *P. aeruginosa* (590), then *K. pneumoniae* (146) and the least in *A. baumannii* strains (133). *P. aeruginosa* isolates shared mostly the non-ribosomal peptide synthase (NRPS) type, *K. pneumoniae* isolates shared the ribosomally synthesized and post-translationally modified peptide-like (RiPP-like) type, while *A. baumannii* isolates shared the siderophore type. Most of the isolates harbored non-ribosomal peptide (NRP) BGCs with few *K. pneumoniae* isolates encoding polyketide BGCs. Sactipeptides and bottromycin BGCs were the most frequently detected RiPP clusters. We hypothesize that each species’ BGC signature confers its virulence. Future experiments will link the detected clusters with their species and determine whether the encoded SMs are produced and cause their virulence.

**IMPORTANCE:**

Our study analyzes the biosynthetic gene clusters (BGCs) present in 66 assemblies from clinical ESKAPE pathogen isolates pertaining to *Acinetobacter baumannii*, *Klebsiella pneumoniae*, and *Pseudomonas aeruginosa* strains. We report their sequencing and assembly followed by the analysis of their BGCs using several bioinformatics tools. We then focused on the most abundant BGC type in each species and we discussed their potential roles in the virulence of each species. This study is pivotal to further build on its experimental work that deciphers the role in virulence, possible antibacterial effects, and characterization of the encoded specialized metabolites (SMs). The study highlights the importance of studying the “harmful” BGCs and understanding the pathogenicity and virulence of those species, as well as possible benefits if the SMs were used as antibacterial agents. This could be the first study of its kind from Egypt and would shed light on BGCs from ESKAPE pathogens from Egypt.

## INTRODUCTION

The ESKAPE term refers to a group of pathogens causing alarming infections in both developed and developing countries due to increasing multidrug resistance and virulence. They include *Enterococcus faecium*, *Staphylococcus aureus*, *Klebsiella pneumoniae*, *Acinetobacter baumanni*, *Pseudomonas aeruginosa*, and *Enterobacter species* (1). According to the 2019 antimicrobial resistance threat report issued by the Centers for Disease Infection and Control (CDC), all members of ESKAPE pathogens are seriously threatening on the nosocomial and community levels in the USA (2). In 2017, the World Health Organization published a priority list of bacterial pathogens for which research and development should be urgently directed to develop new antibiotics. Multidrug-resistant *A. baumanni*, *P. aeruginosa*, and *Enterobacteriaceae* are among the critical pathogens listed for new antibiotic development (3). Thus, all the ESKAPE pathogens group species can be considered superbugs that resist most of the commonly known antibiotics. In addition to being reported as multidrug resistant, ESKAPE pathogens are also reported to be hypervirulent (4).

These pathogens are developing antimicrobial resistance (AMR) against most of the last-line antibiotics such as carbapenems, extended-spectrum beta-lactams (ESBLs), vancomycin, and methicillin (2). Bacterial cells can have intrinsic resistance genes encoded on their chromosomes or can acquire extrachromosomal genes carried on mobile genetic elements such as plasmids and transposons (5). ESBLs are capable of degrading third-generation cephalosporins in addition to penicillin, first- and second-generation cephalosporins, and aztreonam. ESBLs are plasmid-encoded resistance genes and have different types, where the most common are sulfhydryl reagent variable (SHV), TEM, CTX-M, and oxacillinases (OXA) (6). While carbapenem is effective against both Gram-positive and Gram-negative bacteria, carbapenem resistance is a major public threat of infections caused by Gram-negative pathogens. Modifications in penicillin-binding proteins (PBPs), the increase in efflux pumps, and reduction in cell membrane permeability are among the resistance mechanisms to carbapenems (7). Methicillin-resistance *S. aureus* is able to express a variant of PBP, PBP2a, to which methicillin has lower binding affinity (8).

The healthcare system in Egypt is currently struggling with multidrug resistance bacteria in both hospital- and community settings. The misuse and abuse of antibiotics play a key role in the uncontrolled resistance phenomenon. Individuals have easier access to antibiotics as over-the-counter medications instead of being prescribed by healthcare specialists (9). Routine infection control measures in hospitals and reporting resistance cases are cornerstones to systematically tackle AMR issues (10). More effort should be invested to routinely isolate, sequence and study highly prevalent nosocomial and community pathogens and how far or closely related they are compared to their global counterparts.

Some organisms, including bacteria, fungi, and plants, possess in their genomes biosynthetic gene clusters (BGCs) that are encoding for the ultimate synthesis of specialized metabolites (SMs) (11, 12). SMs confer more benefits to the producing organism such as antagonism (e.g., bacteriocins), communication (e.g., homoserine lactones), and survival in harsh environments (e.g., ectoine) (13). SMs are chemically diverse compounds comprising an array of types including polyketides, peptides, terpenes, and others (14). SMs are also known as natural products (NPs, they are encoded in the host genome and are produced by several organisms including pathogens (15, 16).

In addition to the antibiotic activity of a plethora of SMs, as well as SMs of anticancer activity and their use as FDA-approved natural products later on, e.g., erythromycin and doxorubicin, respectively (17); other SMs are contributing to the virulence of their producing organism, e.g., the fungal toxin dihydroxynaphthalene melanin (18).

SMs are more commonly studied and known for their advantageous effects, e.g., as antibacterial agents. Nevertheless, SMs can also have pathogenic effects and can increase the virulence of pathogens producing SMs. Among the examples are *Streptomyces* pathogenic strains that produce geldanamycin and nigericin as SMs and they were reported to have toxic effects on plants (19). It is thus of ultimate importance to study the SMs and their BGCs within pathogenic strains for better understanding and targeting of pathogenicity and virulence of such strains. Other pathogenic strains also were proved to produce SMs that cause harm to their host insects and arthropods (20). SMs were also recently investigated in *P. aeruginosa* clinical isolates as to their metabolites and their structures and were found to produce siderophores, rhamnolipids, quinolones, and phenazines (21). Other examples are *Burkholderia* pathogenic strains and SMs they produce, such as toxoflavin produced by the pathogenic *Burkholderia glumae* (22). It remains interesting to probe pathogenic microbes both for SM culprits and for useful SMs that could possibly be antibacterial against other pathogens (23).

To survive in a hospital setting, ESKAPE pathogens express SMs encoded by BGCs (24). These SMs include antibiotic and anti-biofilm compounds that act against certain bacteria in favor of others (24). On the clinical level, SMs may act in synergism with common AMR mechanisms such as efflux systems and thus render conventional antimicrobials ineffective (25). SMs can also act as antioxidants for reactive oxygen species (ROS), the unstable molecules that contain oxygen and cause cell damage (25). One example of an antioxidant SM is staphyloxanthin produced by *S. aureus* (26). Pyocyanin is a phenazine reduction oxidation SM produced by *P. aeruginosa* as a virulence factor in lung infections (27, 28). Accordingly, SM analysis was suggested to be considered in the standard antibiotic susceptibility tests (25, 29). In *A. baumannii,* wee BGC encodes for extracellular polysaccharide matrix. Targeting this cluster may prevent one of the highest virulence mechanisms of the bacterium, the biofilm (30).

Siderophores are SMs that help bacteria to quench the necessary iron needed for bacterial cells’ growth (31). Normally, host organisms do not have freely moving iron, but the iron is rather tightly bound to proteins. Bacteria can counteract the scarcity of iron by siderophore-dependent and siderophore-independent mechanisms (32). Siderophore production may affect the biofilm formation process in ESKAPE pathogens (31). Siderophores may also interfere with antibiotic activity by modulating oxidative stress mechanisms (33).

With the low-cost and time-efficient sequencing technologies, it is becoming easier to sequence the whole genome of an organism of interest. Whole-genome sequencing has become especially useful with bacteria to mine their genomes and reveal more of their sophisticated metabolic machinery. The therapeutic and industrial potential of BGCs and SMs motivated developers in the microbial bioinformatics field to implement new tools to predict the presence and structure of potential of BGCs within the sequence of bacterial genomes. Bioinformatics and chemoinformatics analysis tools are reviewed in reference (34) in detail.

We aimed to assess the potential of the genomes of selected clinically relevant Gram-negative species pertaining to the species: *K. pneumoniae*, *P. aeruginosa*, and *A. baumannii*, to produce SMs by detection of their potential BGCs. We focused on the clusters that were most abundant in each of the included taxa. The aim of this study was to detect the BGCs of the selected strains and align them with BGCs of known strains. In the future, it is needed to decipher the NPs they produce and their possible roles in the virulence of the strains.

## MATERIALS AND METHODS

### Sample collection, DNA extraction, and sequencing

**Fig 1:**
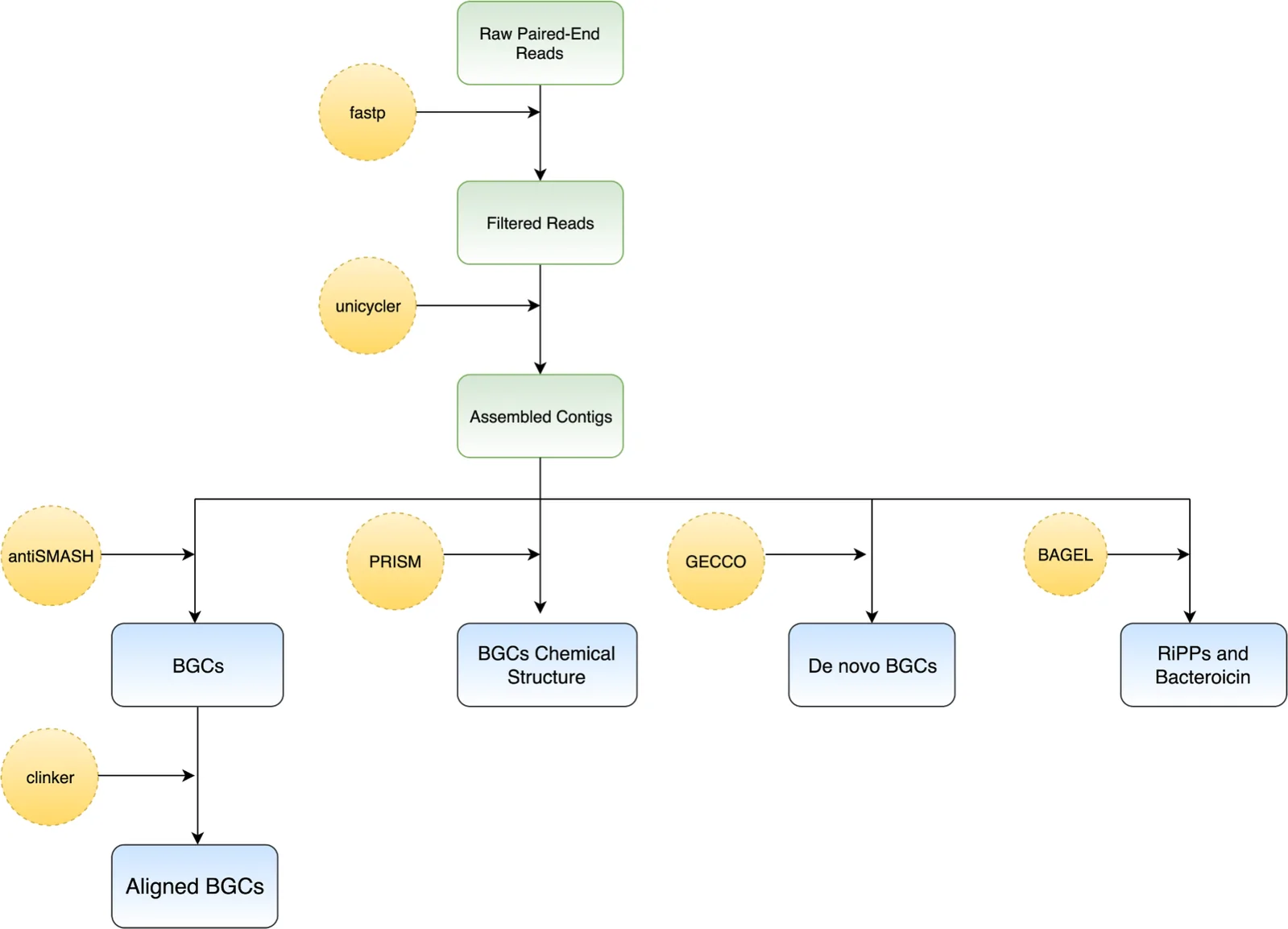
Analysis pipeline workflow.

An overview of the workflow is detailed in Fig. 1. A total of 66 isolates (17 *P. aeruginosa*, 28 *K. pneumoniae*, and 21 *A. baumannii* isolates) were recovered from different clinical specimens (blood, urine, sputum, and others). All samples were randomly collected from patients admitted to bacteriological testing at Mabaret El Asafra Labs, Alexandria, Egypt during the period between August 2020 and March 2021. Bacterial identification at the species level was carried out using the VITEK 2 Compact GN ID card (bioMérieux, Marcy-l’Étoile, France). DNA extraction was performed using QIAamp DNA Mini Kit (QIAGEN) according to the manufacturer’s instructions. DNA quality and concentration were determined using a Qubit 3.0 fluorometer. The Illumina NextSeq 550 sequencing platform was used for whole-genome sequencing of the isolates. For library preparation, 1 μg of genomic DNA and the NEXTflex Rapid XP DNA-Seq library Preparation Kit following the manufacturer’s instructions was used (PerkinElmer, https://perkinelmer-appliedgenomics.com/). The libraries were sequenced using the NextSeq system by NextSeq 500/550 mid output kit v2.5 (300 cycles) paired-end kit.

### Assembly of the draft genomes

Raw paired-end reads of 76 bp length were filtered using fastp (35) with default parameters. Taxonomic classification of the samples was screened using Kmer Finder (36). Identification of strains of all the isolates was concluded using rMLST (37). Three representative strains for each species, based on higher abundance, were included in the analysis. They were PA790 (NZ_CP075176.1), SE5331 (NZ_CP046402.2), and ST773 (NZ_CP041945.1) for *P. aeruginosa,* B12AN (NZ_CABHKQ010000003.1), IR5065 (NZ_CP061948.1), and KpvST147B_SE1_1_NDM (NZ_CP040724.1) for *K. pneumoniae*, and ACN21 (NZ_CP038644.1), MS14413 (NZ_CP054302.1), and TP3 (NZ_CP060013.1) for *A. baumannii,* respectively. Complete FASTA files were downloaded for these representative strains from RefSeq database (38). Filtered reads were assembled using Unicycler software (39). Contigs assembly quality was assessed using QUAST (40). The metadata of the assembled contigs is detailed in Table S1 at https://github.com/lailaziko/Gram-Negative-BGCs.

### Detection of BGCs

**Fig 2 F2:**
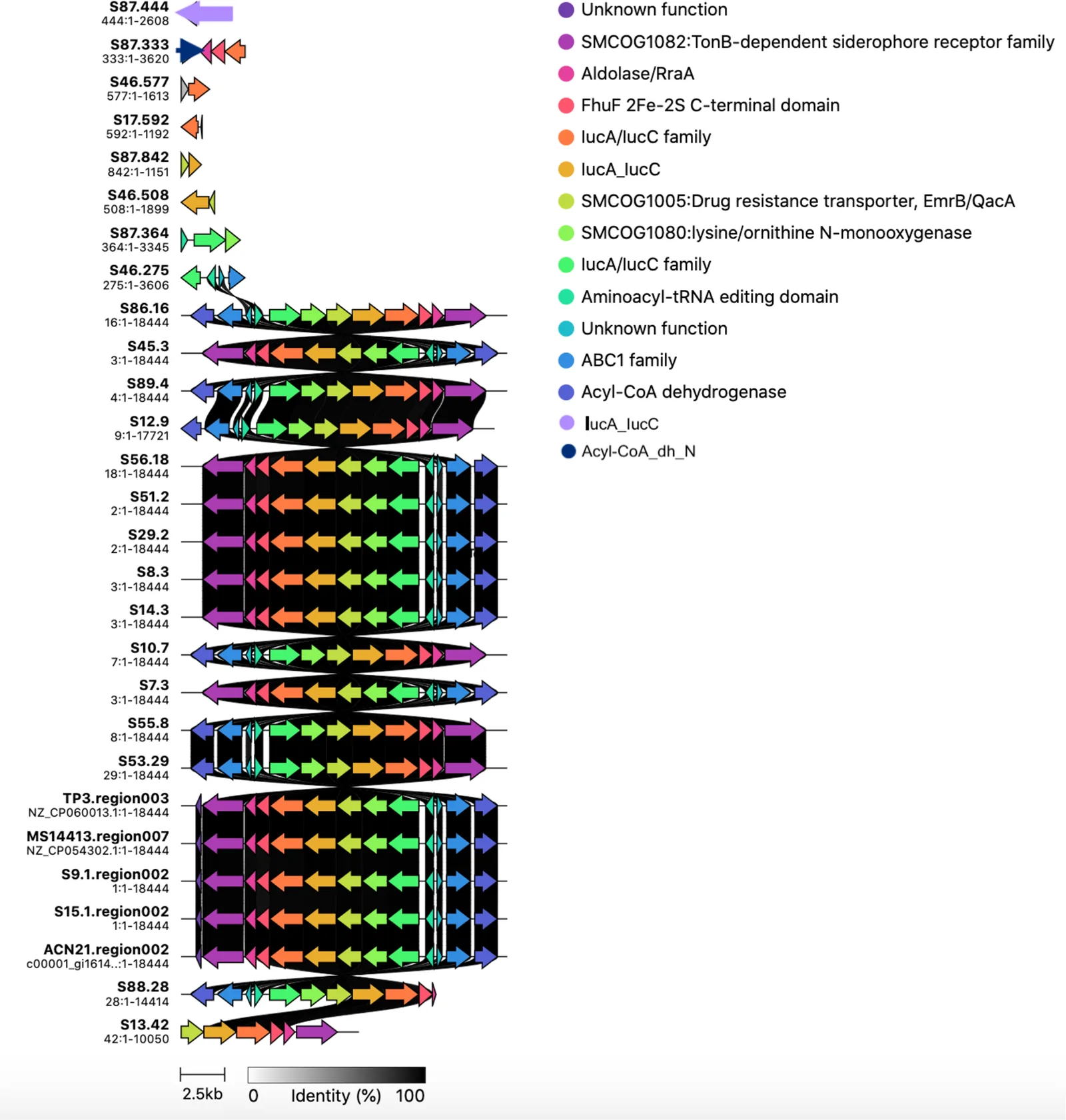
Siderophore BGCs in *Acinetobacter baumannii.* The colors in the legend indicate the biosynthetic gene clusters of interest. Other colors are either of unknown function or not the main aim of the current study. The letter “S” stands for the sample, followed by the sample number, then the contig number, then the base pair position of the BGC on this contig.

**Fig 3 F3:**
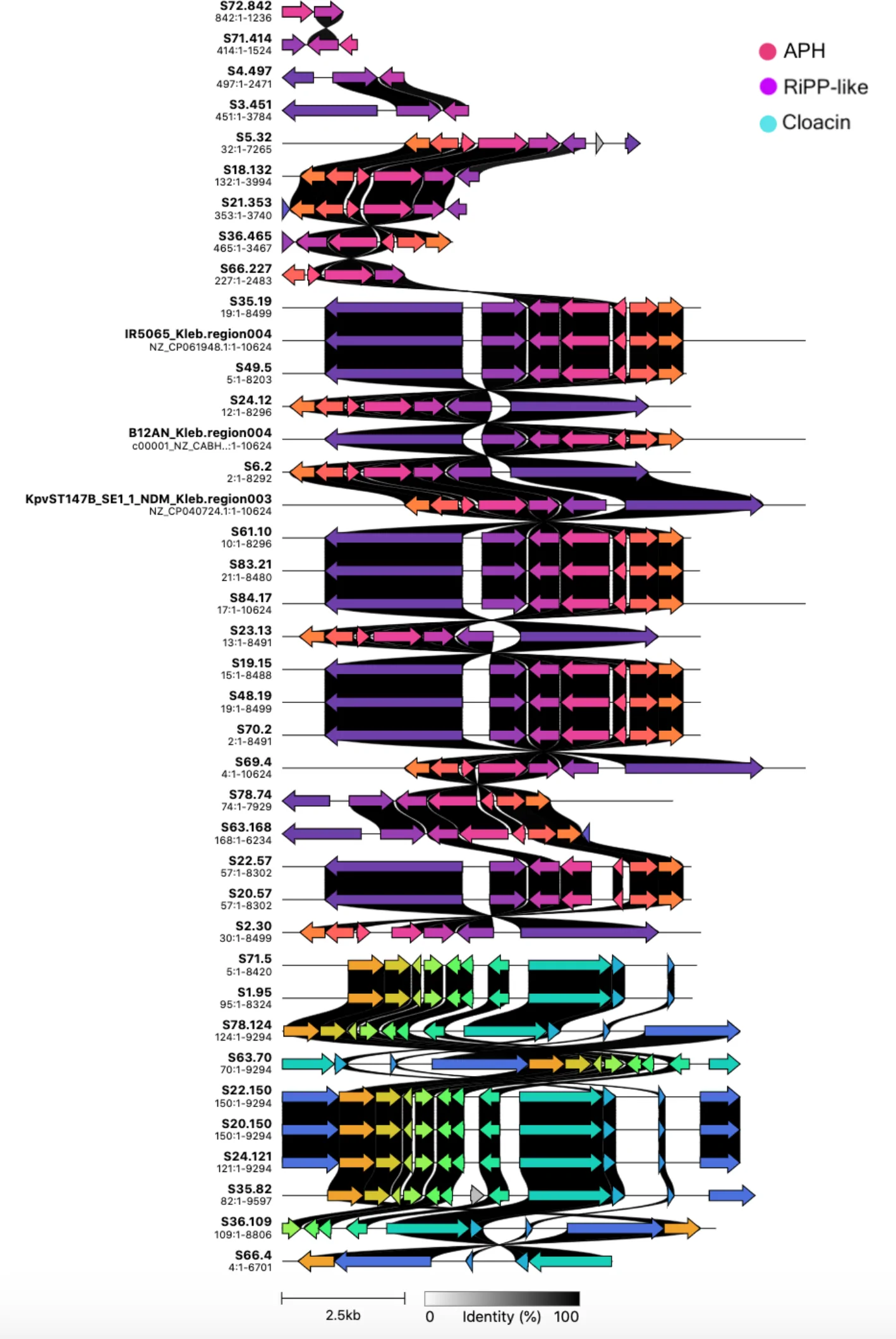
RiPP-like BGCs in *Klebsiella pneumoniae.* Colors that are not present in the legend represent gene clusters of functions other than BGCs.

**Fig 4 F4:**
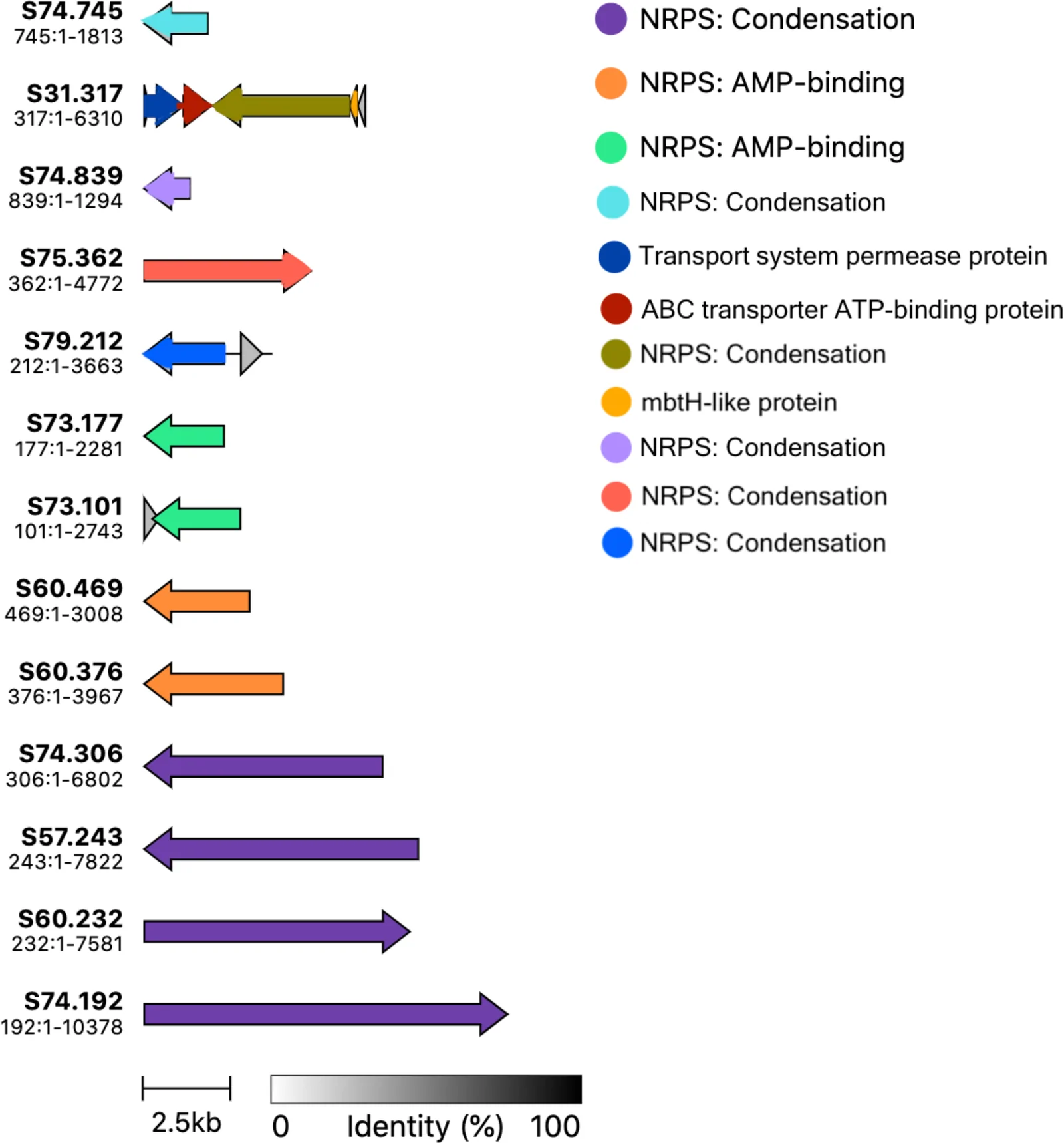
NRPS BGC subgroup 1 in *Pseudomonas aeruginosa*. Other NRPS subgroups in *Pseudomonas aeruginosa* isolates are grouped according to alignment and similarity and provided in the figures at https://github.com/lailaziko/Gram-Negative-BGCs.

We used four bioinformatics tools for BGCs detection and analysis. The first one was antiSMASH software (v6.0.1), where detection strictness was set into relaxed (14). Algorithms used for BGCs searching were Known Cluster Blast, Active Site Binder, SubCluster Blast, and RREFinder. Selected BGCs were as follows: non-ribosomal peptide synthase (NRPS), RiPP-like, and siderophores, as they were the most abundant BGCs found in *P. aeruginosa*, *K. pneumoniae*, *and A. baumannii*, respectively. Using GenBank files generated from antiSMASH, the selected BGCs in the samples and representative strains were visualized using command-line Clinker software (41) where alignment was included, and the identity was set to 90%, clusters were labeled according to gene functions, and similar clusters were linked. A spectrum of colors was set for each gene cluster but only clusters of interest were annotated in the figure legends of Fig. 2 to 4; Fig. S1 to S8 at https://github.com/lailaziko/Gram-Negative-BGCs. As *P. aeruginosa* showed multiple NRPS regions in all the samples, Clinker presentation of NRPS was divided on multiple panels (Fig. 4; Fig. S1 to S8 at https://github.com/lailaziko/Gram-Negative-BGCs). Clinker automatically assigns colors for homologous genes as provided by GenBank files output from antiSMASH software. As samples for each species were analyzed independently, different colors might be assigned. Therefore, a detailed color legend for each figure was provided. Some samples encoded similar BGCs but not homologous—i.e., not aligning—for Clinker to assign them all the same color. To further check Clinker output, we manually curated the BGC Genbank files generated by antiSMASH to match with Clinker and gray-colored clustered were re-colored if they presented BGCs of interest.

To infer the chemical structure of the selected BGCs and further detect other putative BGCs, PRISM software (42) was utilized with default parameters used for the analysis needed. GECCO was used to (43) analyze the assembled contigs for *de novo* BGCs and BAGEL was employed (44) for RiPPs and bacteriocin detection.

### Data analysis

R was used for the hierarchical classification of the detected BGCs. The normalization was done by dividing each number by the genome size and multiplied it by 10^6^. The heatmap3 package was used for the generation of the heatmaps (45).

## RESULTS

### BGCs in *P. aeruginosa* draft genomes

**Fig 5 F5:**
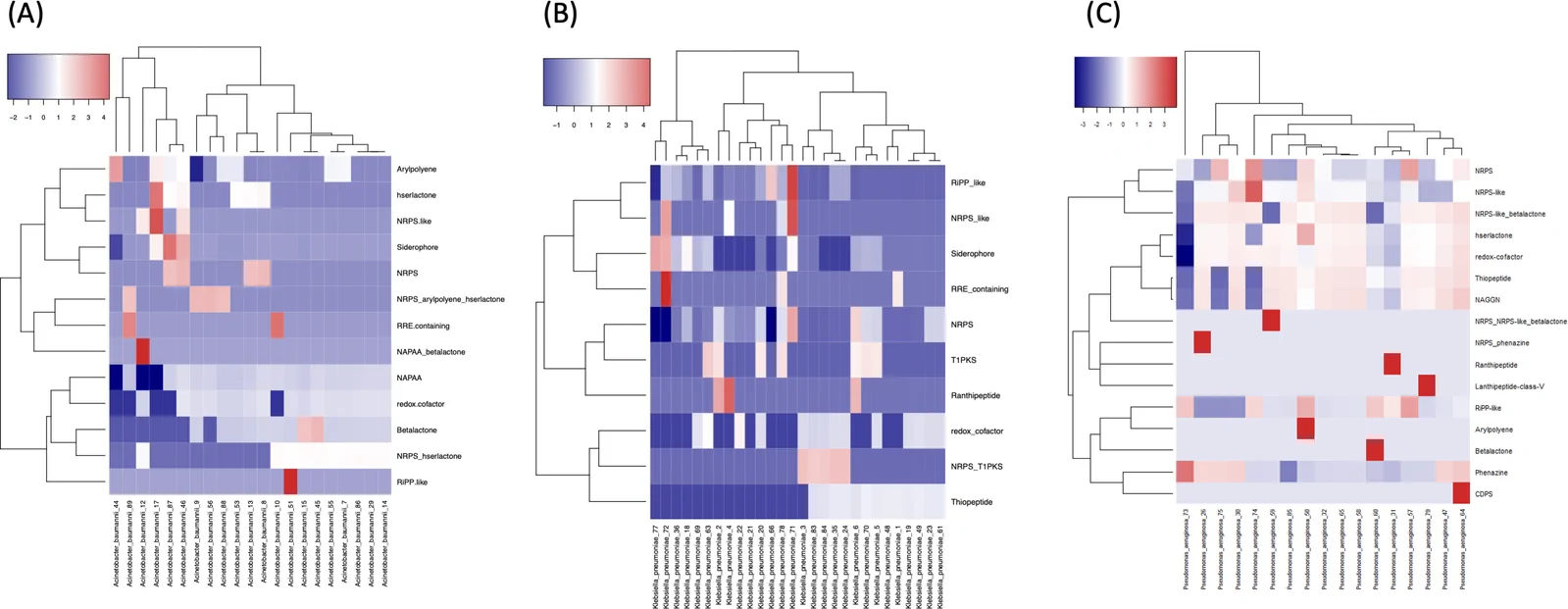
Hierarchical clustering of the bacteria based on their BGC profiles: (**A**) *Acinetobacter baumannii*, (**B**) *Klebsiella pneumoniae*, and (**C**) *Pseudomonas aeruginosa*.

In total, 590 BGCs were detected by antiSMASH in all the included genomes. Among those, 311 BGCs were detected in the included *P. aeruginosa* genomes. The most abundant BGC class was NRPS BGCs (117) in all screened isolates, also RiPP-like BGCs were detected in all 17 samples (39). The unique BGCs were NRPS-like_betalactone, Phenazine, NRPS_phenazine, N-acetylglutaminylglutamine amide (NAGGN), NRPS_NRPS-like_betalactone, and CDPS BGCs. All the detected normalized BGCs are shown in the heatmap (Fig. 5C). The detailed BGCs detected by antiSMASH are in Table S2 at https://github.com/lailaziko/Gram-Negative-BGCs. We selected the NRPS BGCs to display the alignment of the detected BGCs as per Clinker visualization tool (Fig. 4; Fig. S1 to S8 at https://github.com/lailaziko/Gram-Negative-BGCs [Fig F7]).

**Fig 6 F6:**
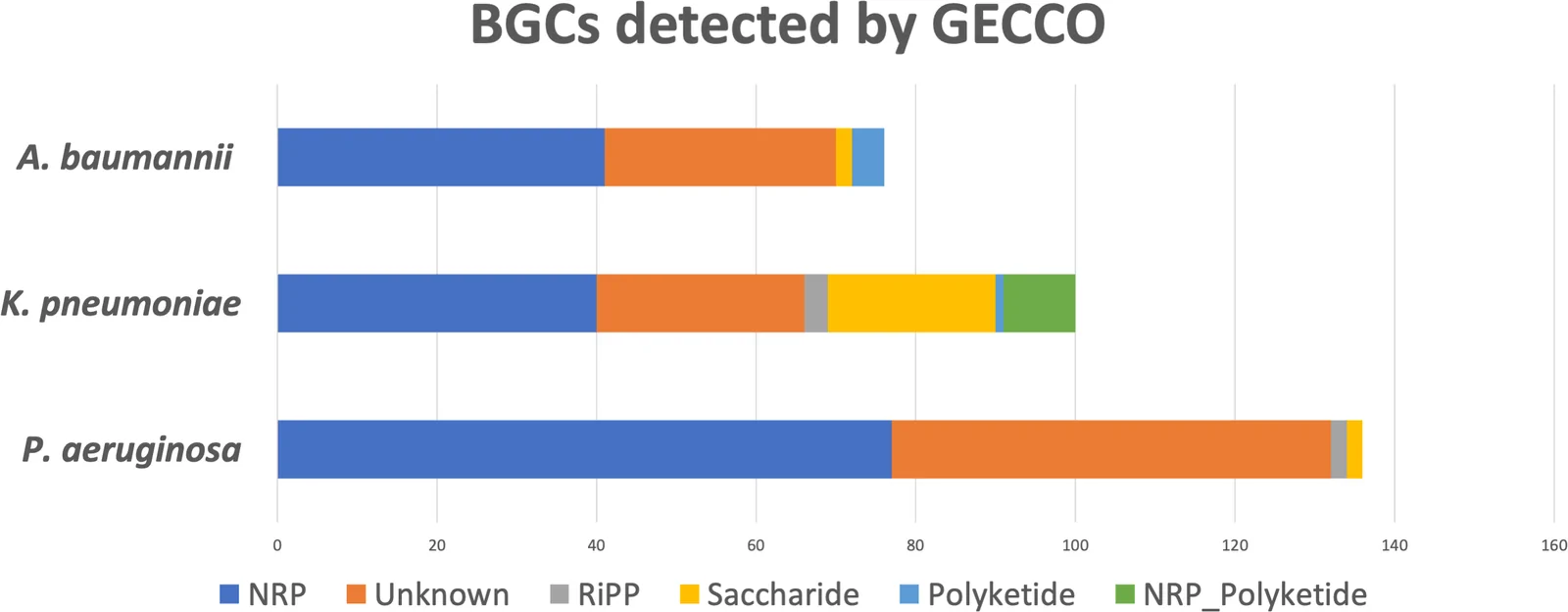
GECCO results for all isolates of each species.

**Fig 7 F7:**
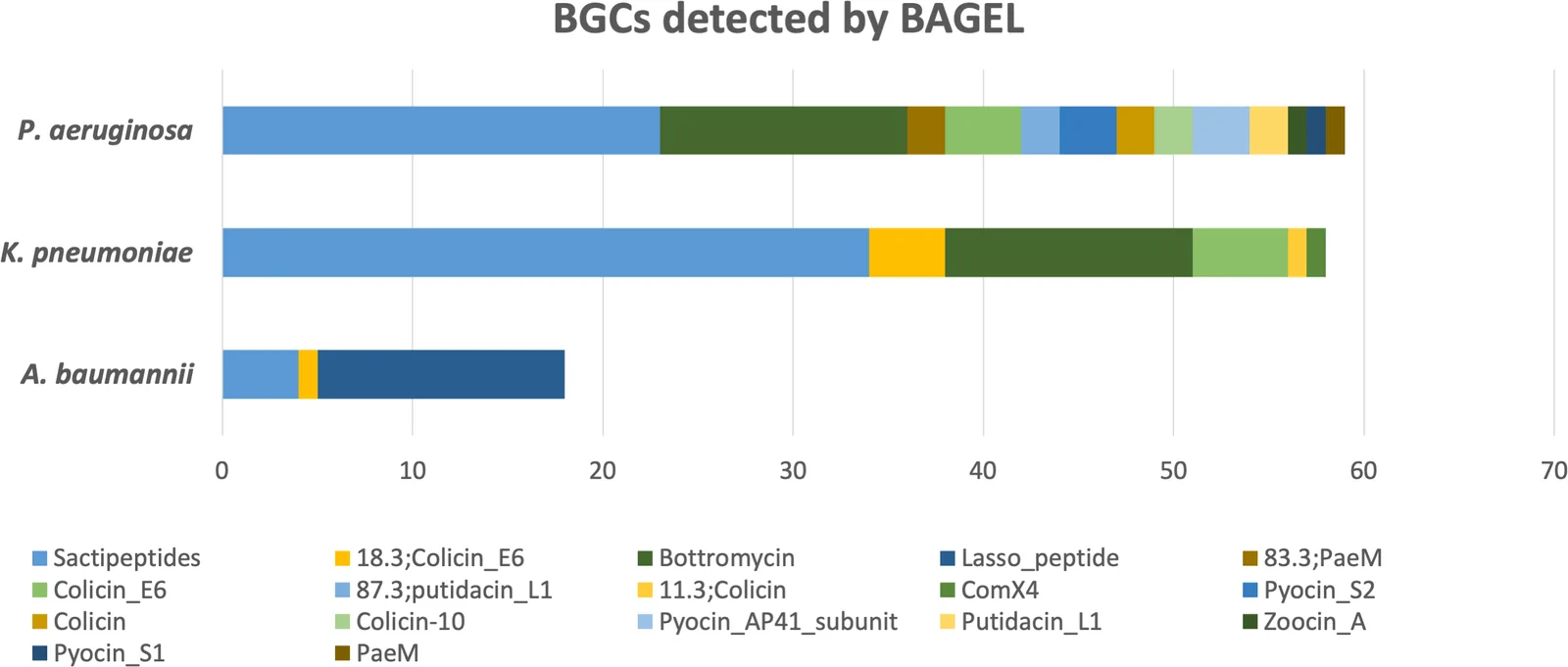
BAGEL results for all isolates of each species.

GECCO detected 136 BGCs in *P. aeruginosa* genomes, with non-ribosomal peptides (NRPs) (77), Unknown (55), RiPP (2), and Saccharide (2) BGCs (Fig. 6 [Fig F6]; Fig. S9 at https://github.com/lailaziko/Gram-Negative-BGCs). PRISM detected 209 BGCs detailed in Table S3 at https://github.com/lailaziko/Gram-Negative-BGCs, and 178 structures were predicted. BAGEL revealed a total of 59 hits with *P. aeruginosa* isolates, and they were as follows (Fig. 7; Fig. S10 at https://github.com/lailaziko/Gram-Negative-BGCs): sactipeptides (23), bottromycin (13), 83.3;PaeM (2), colicin_E6 (4), 87.3;putidacin_L1 (2), pyocin_S2 (3), colicin (2), colicin-10 (2), pyocin_AP41_subunit (3), putidacin_L1 (2), zoocin_A (1), pyocin_S1 (1), and PaeM (1).

### BGCs in *A. baumannii* draft genomes

A total of 133 BGCs were detected in the *A. baumannii* genomes. The most abundant BGC classes were Arylpolyene (26) and Siderophore (25) BGCs in 20 of the 21 screened *A. baumannii* isolates. The unique BGCs were NRPS_hserlactone (10), NAPAA (18), and NAPAA_betalactone (1). All the detected normalized BGCs are shown in the heatmap (Fig. 5A). The detailed BGCs detected by antiSMASH are in Table S2 at https://github.com/lailaziko/Gram-Negative-BGCs.

We selected the siderophore BGCs to display the alignment of the detected BGCs as per Clinker visualization tool (Fig. 2). GECCO detected 76 BGCs in *A. baumannii*, genomes, with NRP (41), Unknown (29), Saccharide (2), and Polyketide (4) BGCs (Fig. 6; Fig. S9 at https://github.com/lailaziko/Gram-Negative-BGCs). PRISM detected 70 clusters detailed in Table S3 at https://github.com/lailaziko/Gram-Negative-BGCs, and 45 structures were predicted. BAGEL revealed the least hits with *A. baumannii* isolates (Fig. 7; Fig. S10 at https://github.com/lailaziko/Gram-Negative-BGCs), with a total of 18 hits, and they were mainly lassopeptides (13), sactipeptides (4), and 18.3;Colicin_E6 (1).

### BGCs in *K. pneumoniae* draft genomes

A total of 146 BGCs were detected in the *K. pneumoniae* genomes. The most abundant BGC class was RiPP-like (35) in 27 of the 28 screened isolates. The unique BGCs were T1PKS (7) and NRPS_T1PKS (5) BGCs. All the detected normalized BGCs are shown in the heatmap (Fig. 5B). The detailed BGCs detected by antiSMASH are shown in Table S2 at https://github.com/lailaziko/Gram-Negative-BGCs.

We selected the RiPP-like BGCs to display the alignment of the detected BGCs as per Clinker visualization tool (Fig. 3). GECCO detected 100 BGCs in *K. pneumoniae* genomes, with NRP (40), Unknown (26), RiPP (3), Saccharide (21), Polyketide (1), and NRP_Polyketide (9) BGCs (Fig. 6; Fig. S9 at https://github.com/lailaziko/Gram-Negative-BGCs). PRISM detected 87 clusters detailed in Table S3 at https://github.com/lailaziko/Gram-Negative-BGCs, and 47 structures were predicted. BAGEL revealed a total of 58 hits with *K. pneumoniae* isolates, and they were as follows (Fig. 7 and S10 at https://github.com/lailaziko/Gram-Negative-BGCs): sactipeptides (34), bottromycin (13), Colicin_E6 (5), 18.3;Colicin_E6 (4), 11.3;Colicin (one cluster), and ComX4 (1).

## DISCUSSION

### NRPS BGCs in *P. aeruginosa* draft genomes

Three NRPS BGCs are well-characterized in *P. aeruginosa* species standard strain PA01, and they encode for the formation of pyoverdine (the siderophore key player associated with pathogenesis), pyochelin (a siderophore produced by *P. aeruginosa*), L-2-Amino-4-methoxy-*trans*-3-butenoic acid (AMB) (involved in quorum sensing), pyoluteorin (an antibacterial compound), in addition to three uncharacterized NRPS BGCs (46). Recently, a mimic of an NRPS pathway to produce mimics of the antibiotic brevicidine was changed to be a ribosomal pathway instead, and this highlights the importance of studying NRPSs and their products as antibacterial agents (47). Further analysis of the NRPS clusters in the strains included in this study is required, and the comparison with the well-characterized *Pseudomonas* NRPS BGCs, as well as unraveling their function, whether in their contribution to the strain’s virulence or to their possible application as antibacterial agents. The results from GECCO were aligning with the antiSMASH results for the *P. aeruginosa* genomes, as indeed the largest detected class was NRP and it was higher than those also detected in the genomes pertaining to *K. pneumoniae* and *A. baumannii*. This warrants further studying, and interestingly, the unknown clusters were the second largest class and require further studying as to what is being coded for, and whether they are cryptic or active BGCs. In general, for all the included strains, antiSMASH detected more BGCs than GECCO platform. Although the difference was most prominent with *P. aeruginosa* genomes (590 vs 136 BGCs), and the least with *K. pneumoniae* genomes (146 vs 100 BGCs), it could possibly be because of the inclusion of more BGC types in those strains included in the database of antiSMASH.

The hits obtained by BAGEL are worth pursuing experimentally. Sactipeptides and bottromycin warrant further experimental studying, as well as the unique clusters that were detected uniquely in isolates pertaining to this species. Sactipeptides were recently found to be produced by *Streptomyces thermophilus* strain and one—streptosactin—was recently characterized and exhibited antibacterial activity (48). Bottromycin is known for its antibacterial effect (49), however, perhaps finding similarity with it would lead to an SM that is antagonistic and in this context, contributing to the virulence of the strains. Perhaps because *P. aeruginosa* strains are predicted to encode primarily peptides, they had also the most detected hits in BAGEL, as it is a database specific for detection of bacteriocins and RiPPs (50). PaeM is a bacteriocin produced by *P. aeruginosa* strains (51), and it is worth investigating the hit in the included strains, and testing them for antibacterial effects. The detected pyocins are particularly interesting hits as they are reported bacteriocin types that assist the formation of biofilms and hence are virulence factors (52). Zoocin is also a bacteriocin (53), and it was detected in the included samples. Putidacin is a bacteriocin that is of the type of lectin-like (54). Colicins are bacteriocins that were studied in *Escherichia coli* (52), and their detection in this species is also worth pursuing.

The NRPS clusters abundant in *P. aeruginosa* isolates are depicted in Fig. 4; Fig. S1 to S8 at https://github.com/lailaziko/Gram-Negative-BGCs. They are mainly distributed among nine panels according to the alignment and are important for further comparisons, and it shows how there were different NRPS BGCs and thus were aligned differently. We attempted to group together the *P. aeruginosa* isolates for similar BGC signature profiles (Fig. 5C), and the closer strains cluster together in the heatmap into five main clusters. Table S3 at https://github.com/lailaziko/Gram-Negative-BGCs includes all the details about the hits obtained by PRISM, and 178 structures were predicted, some were basic structures while some were more detailed chemical structures. We herein report them as a lead together with their annotation in order to be used later for experimental analysis, they include mainly non-ribosomal peptides and acyl homoserine lactones, among other classes.

### RiPP-like BGCs in *K. pneumoniae* draft genomes

RiPP-like clusters are detected by antiSMASH and comprise BGCs that are not detected as RiPPs but are however found regularly with RiPPs, including bacteriocins and other unspecified RiPPs (14). Bacteriocins have antibacterial activities and are peptidic in nature that are ribosomally synthesized (55). Bacteriocins were recently detected in *Klebsiella* genus and identified as klebicins, which are colicin-like bacteriocins (56). These klebicins were found to be effective against *Klebsiella* clinical isolates, in support that actually bacteriocins are effective against members of the ESKAPE pathogens (56). GECCO results were not in concordance with the antiSMASH results in this regard, as the majority of the detected BGCs comprised NRPs, followed by unknown clusters; however, this discrepancy could be explained by the naming each platform uses, in addition to the inherent workflow difference, that renders GECCO capable of predicting novel BGCs, rather than aligning with characterized BGCs. Interestingly, *K. pneumoniae* isolates harbored one putative polyketide and nine NRP_polyketide clusters, which were particularly unique and warrants further experimental validation. Future work on our data would encompass that the bacteriocin sequences are further analyzed and tested as to their potential antimicrobial effect.

The hits retrieved from BAGEL were close to the antiSMASH results, as sactipeptides were detected. Their role in pathogenicity as well as targeting them by genus-specific drugs in the future are worth investigating. Bottromycin hits need also further studying, as well as the uniquely detected hits. ComX4 is a RiPP that is involved in surfactin synthesis by being a quorum-sensing player (57), and should be investigated for its role in the included *K. pneumoniae* genomes. Sactipeptide, bottromycin, and colicin BGCs were also recently reported in clinical isolates of *Klebsiella* in Thailand (58).

The RiPP-like BGCs which were most common among the *K. pneumoniae* genomes were aligned and are depicted in Fig. 3, with the similar BGCs aligned together. Among the detected clusters such as RiPP-like cloacin, which is a bacteriocin and coincides in its class with the BAGEL results (59). RiPP-like TIGR03651 was also detected, which belongs to the circular bacteriocin, circularin A/uberolysin family (60). Biosynthetic aminoglycoside phosphotransferase (APH) genes were found, which were earlier reported in the biosynthesis of thiostreptamide S4 which belongs to the anticancer class of compounds, the thioamitide class (61), and hence warrants further investigation. We attempted to group together the *K. pneumoniae* isolates for similar BGC signature profiles (Fig. 2B), and the closer strains cluster together in the heatmap into four main clusters. Table S2 at https://github.com/lailaziko/Gram-Negative-BGCs includes all the details about the hits obtained by PRISM, and 51 structures were predicted, some were basic structures, and some were more detailed chemical structures. We herein report them as a lead together with their annotation in order to be used later for experimental analysis, they include mainly non-ribosomal peptides, polyketides, NRPS-independent siderophore synthases, and polyketide-non-ribosomal peptides, among other classes.

### Siderophore and aryl polyene BGCs in *A. baumannii* draft genomes

Siderophore BGCs were recently detected in *Nocardia* species and possibly contributing to their pathogenicity (62). Recently it was found that *A. baumannii* utilize several siderophores mainly to bind iron and hence are harboring multiple siderophore BGCs, and one particular siderophore is pertaining to its virulence, namely acinetobactin (63). There are up to 10 siderophore BGCs within *A. baumannii* genomes (63). The included strains harbored siderophore BGCs in common and their alignment with known *A. baumannii* BGCs remains to be investigated, as well as their roles in the strain virulence. Aryl polyene BGCs were detected within the genome of the virulent *Acinetobacter* strain global clone 2 (GC2) (64). Aryl polyene BGCs code for the production of 4-hydroxybenzoyl polyene compounds, and they have a hypothesized function to escape the host immune system (64). The aryl polyene BGCs detected in this study require further analysis as to their similarity to characterized aryl polyene BGCs of other strains and their role to be investigated. The GECCO results were not in concordance with the antiSMASH detected BGCs, as the most BGCs were detected as NRPs, which could possibly be because of the different classes detected by each tool, and that GECCO types are more limited than antiSMASH BGC types that could be detected. It is noteworthy that 29 unknown BGCs were detected as well as four polyketides, which points toward their study and might explain those detected by antiSMASH as well as additional ones.

It is noteworthy that the lasso peptides are prominent in this genus, as well as the unique clusters detected. The functions of the detected clusters need to be deciphered, as to their role in virulence and could be possible targets for drugs towards these specific strains. Lasso peptides were earlier produced by other *Acinetobacter* strains, such as *A. gyllenbergii*, that produce acinetodin (65). Sactipeptides and colicins are also bacteriocins as mentioned earlier, and their role in pathogenicity as well as possible antibacterial effects need to be further investigated.

The siderophores were most commonly detected in *A. baumannii* genomes and their alignment is depicted in Fig. 2. lucA//lucC gene family hits were detected in the siderophore BGCs as it is an iron uptake chelate domain involved in siderophores biosynthesis. lucA and lucC are ligases and NRPS-independent siderophore synthetases that were previously studied in hypervirulent *K. pneumoniae* (66). We attempted to group together the *A. baumannii* genomes for similar BGC signature profiles (Fig. 5A), and the closer strains cluster together in the heatmap into two main clusters. Table S3 at https://github.com/lailaziko/Gram-Negative-BGCs includes all the details about the hits obtained by PRISM, and 45 structures were predicted, some were basic structures, and some were more detailed chemical structures. We herein report them as a lead together with their annotation to be used later for experimental analysis, they include mainly non-ribosomal peptides, NRPS-independent siderophore synthases, acyl homoserine lactone-non-ribosomal peptide, and acyl homoserine lactones, among other classes.

In conclusion, our study investigates the BGCs present in three members of the ESKAPE pathogen panel that are relevant in hospitals and has highlighted the most common BGC type in each of the *A. baumannii*, *K. pneumoniae*, and *P. aeruginosa* strains. We predict those BGCs perhaps play a specific role in the virulence of each strain, which warrants further experimental validation. Several isolates of the same species were analyzed to study the similarities and differences between their encoded BGCs. The BGCs pertaining to the same species indeed show differences as visualized in Fig. 2 to 7;[Fig F2 F3 F6 F7 F4 F5] [Fig F2 F3 F6 F7 F4 F5] Fig. S1 to S8 at https://github.com/lailaziko/Gram-Negative-BGCs, with the common and different BGCs indicated. There is a common signature BGC profile for each species; however, there were inter-species differences with regard to their BGCs.

## Data Availability

The raw reads were deposited in NCBI under the Bioprojects: PRJNA906142, PRJNA906139, and PRJNA906141 for *P. aeruginosa*, *K. pneumoniae*, and *A. baumannii*, respectively. The accession numbers of the representative strains used are as aforementioned. The supplementary files are available online at: https://github.com/lailaziko/Gram-Negative-BGCs.

## References

[1] Rice LB . 2008. Federal funding for the study of antimicrobial resistance in nosocomial pathogens: no ESKAPE. J Infect Dis 197:1079–1081. doi:10.1086/533452 18419525

[2] Antibiotic resistance threats in the United States. 2019. Centers for Disease Control and Prevention. [cited 2022 Dec 12]. Available from: https://www.cdc.gov/drugresistance/pdf/threats-report/2019-ar-threats-report-508.pdf

[3] WHO publishes list of bacteria for which new antibiotics are urgently needed [Internet]. World Health Organization (WHO). [cited 2022 Dec 12]. Available from: https://www.who.int/news/item/27-02-2017-who-publishes-list-of-bacteria-for-which-new-antibiotics-are-urgently-needed

[4] De Oliveira DMP , Forde BM , Kidd TJ , Harris PNA , Schembri MA , Beatson SA , Paterson DL , Walker MJ . 2020. Antimicrobial resistance in ESKAPE pathogens. Clin Microbiol Rev 33:e00181-19. doi:10.1128/CMR.00181-19 32404435 PMC7227449

[5] Darby EM , Trampari E , Siasat P , Gaya MS , Alav I , Webber MA , Blair JMA . 2023. Molecular mechanisms of antibiotic resistance revisited. Nat Rev Microbiol 21:280–295. doi:10.1038/s41579-022-00820-y 36411397

[6] Paterson DL , Bonomo RA . 2005. Extended-spectrum β-lactamases: a clinical update. Clin Microbiol Rev 18:657–686. doi:10.1128/CMR.18.4.657-686.2005 16223952 PMC1265908

[7] Zapun A , Contreras-Martel C , Vernet T . 2008. Penicillin-binding proteins and β-lactam resistance. FEMS Microbiol Rev 32:361–385. doi:10.1111/j.1574-6976.2007.00095.x 18248419

[8] Stapleton PD , Taylor PW . 2002. Methicillin resistance in Staphylococcus aureus: mechanisms and modulation. Sci Prog 85:57–72. doi:10.3184/003685002783238870 11969119 PMC2065735

[9] Sabry NA , Farid SF , Dawoud DM . 2014. Antibiotic dispensing in Egyptian community pharmacies: an observational study. Res Social Adm Pharm 10:168–184. doi:10.1016/j.sapharm.2013.03.004 23665078

[10] Wall S . 2019. Prevention of antibiotic resistance – an epidemiological scoping review to identify research categories and knowledge gaps. Glob Health Action 12:1756191. doi:10.1080/16549716.2020.1756191 32475304 PMC7782542

[11] Jensen PR , Chavarria KL , Fenical W , Moore BS , Ziemert N . 2014. Challenges and triumphs to genomics-based natural product discovery. J Ind Microbiol Biotechnol 41:203–209. doi:10.1007/s10295-013-1353-8 24104399 PMC3946964

[12] Jensen PR . 2016. Natural products and the gene cluster revolution. Trends Microbiol 24:968–977. doi:10.1016/j.tim.2016.07.006 27491886 PMC5123934

[13] Junkins EN , McWhirter JB , McCall L-I , Stevenson BS . 2022. Environmental structure impacts microbial composition and secondary metabolism. ISME Commun 2. doi:10.1038/s43705-022-00097-5 PMC9723690 37938679

[14] Blin K , Shaw S , Kloosterman AM , Charlop-Powers Z , van Wezel GP , Medema MH , Weber T . 2021. antiSMASH 6.0: improving cluster detection and comparison capabilities. Nucleic Acids Res 49:W29–W35. doi:10.1093/nar/gkab335 33978755 PMC8262755

[15] Davies J . 2013. Specialized microbial metabolites: functions and origins. J Antibiot (Tokyo) 66:361–364. doi:10.1038/ja.2013.61 23756686

[16] Davies J , Ryan KS . 2012. Introducing the parvome: bioactive compounds in the microbial world. ACS Chem Biol 7:252–259. doi:10.1021/cb200337h 22074935

[17] Newman DJ , Cragg GM . 2020. Natural products as sources of new drugs over the nearly four decades from 01/1981 to 09/2019. J Nat Prod 83:770–803. doi:10.1021/acs.jnatprod.9b01285 32162523

[18] Scharf DH , Heinekamp T , Brakhage AA . 2014. Human and plant fungal pathogens: the role of secondary metabolites. PLoS Pathog 10:e1003859. doi:10.1371/journal.ppat.1003859 24497825 PMC3907374

[19] Díaz-Cruz GA , Liu J , Tahlan K , Bignell DRD . 2022. Nigericin and geldanamycin are phytotoxic specialized metabolites produced by the plant pathogen Streptomyces sp. 11-1-2. Microbiol Spectr 10:e0231421. doi:10.1128/spectrum.02314-21 35225656 PMC9045263

[20] Mollah MMI , Kim Y . 2020. Virulent secondary metabolites of entomopathogenic bacteria genera, xenorhabdus and photorhabdus, inhibit phospholipase A2 to suppress host insect immunity. BMC Microbiol 20:359. doi:10.1186/s12866-020-02042-9 33228536 PMC7684946

[21] Lybbert AC , Williams JL , Raghuvanshi R , Jones AD , Quinn RA . 2020. Mining public mass spectrometry data to characterize the diversity and ubiquity of P. aeruginosa specialized metabolites. Metabolites 10:445. doi:10.3390/metabo10110445 33167332 PMC7694397

[22] Elshafie HS , Camele I . 2021. An overview of metabolic activity, beneficial and pathogenic aspects of Burkholderia Spp. Metabolites 11:321. doi:10.3390/metabo11050321 34067834 PMC8156019

[23] Maglangit F , Yu Y , Deng H . 2021. Bacterial pathogens: threat or treat (a review on bioactive natural products from bacterial pathogens). Nat Prod Rep 38:782–821. doi:10.1039/d0np00061b 33119013

[24] Tiwari V , Meena K , Tiwari M . 2018. Differential anti-microbial secondary metabolites in different ESKAPE pathogens explain their adaptation in the hospital setup. Infect Genet Evol 66:57–65. doi:10.1016/j.meegid.2018.09.010 30227225

[25] Perry EK , Meirelles LA , Newman DK . 2022. From the soil to the clinic: the impact of microbial secondary metabolites on antibiotic tolerance and resistance. Nat Rev Microbiol 20:129–142. doi:10.1038/s41579-021-00620-w 34531577 PMC8857043

[26] Clauditz A , Resch A , Wieland K-P , Peschel A , Götz F . 2006. Staphyloxanthin plays a role in the fitness of Staphylococcus aureus and its ability to cope with oxidative stress. Infect Immun 74:4950–4953. doi:10.1128/IAI.00204-06 16861688 PMC1539600

[27] Lau GW , Hassett DJ , Ran H , Kong F . 2004. The role of pyocyanin in Pseudomonas aeruginosa infection. Trends Mol Med 10:599–606. doi:10.1016/j.molmed.2004.10.002 15567330

[28] Zhu K , Chen S , Sysoeva TA , You L , Balaban N . 2019. Universal antibiotic tolerance arising from antibiotic-triggered accumulation of pyocyanin in Pseudomonas aeruginosa. Edited by N Balaban . PLoS Biol 17:e3000573. doi:10.1371/journal.pbio.3000573 31841520 PMC6936868

[29] Meirelles LA , Newman DK . 2022. Phenazines and toxoflavin act as interspecies modulators of resilience to diverse antibiotics. Mol Microbiol 117:1384–1404. doi:10.1111/mmi.14915 35510686 PMC10249331

[30] Tiwari M , Panwar S , Kothidar A , Tiwari V . 2020. Rational targeting of Wzb phosphatase and Wzc kinase interaction inhibits extracellular polysaccharides synthesis and biofilm formation in Acinetobacter baumannii. Carbohydr Res 492:108025. doi:10.1016/j.carres.2020.108025 32402850

[31] Post SJ , Shapiro JA , Wuest WM . 2019. Connecting iron acquisition and biofilm formation in the ESKAPE pathogens as a strategy for combatting antibiotic resistance. Medchemcomm 10:505–512. doi:10.1039/c9md00032a 31057729 PMC6482887

[32] Tiwari V . 2013. Effect of iron availability on the survival of carbapenem-resistant Acinetobacter baumannii: a proteomic approach. J Proteomics Bioinform 06:06. doi:10.4172/jpb.1000270

[33] Lazar V , Holban AM , Curutiu C , Chifiriuc MC . 2021. Modulation of quorum sensing and biofilms in less investigated gram-negative ESKAPE pathogens. Front Microbiol 12:676510. doi:10.3389/fmicb.2021.676510 34394026 PMC8359898

[34] Kim HU , Blin K , Lee SY , Weber T . 2017. Recent development of computational resources for new antibiotics discovery. Curr Opin Microbiol 39:113–120. doi:10.1016/j.mib.2017.10.027 29156309

[35] Chen S , Zhou Y , Chen Y , Gu J . 2018. fastp: an ultra-fast all-in-one FASTQ Preprocessor. Bioinformatics 34:i884–i890. doi:10.1093/bioinformatics/bty560 30423086 PMC6129281

[36] Clausen P , Aarestrup FM , Lund O . 2018. Rapid and precise alignment of raw reads against redundant databases with KMA. BMC Bioinformatics 19:307. doi:10.1186/s12859-018-2336-6 30157759 PMC6116485

[37] Jolley KA , Bliss CM , Bennett JS , Bratcher HB , Brehony C , Colles FM , Wimalarathna H , Harrison OB , Sheppard SK , Cody AJ , Maiden MCJ . 2012. Ribosomal multilocus sequence typing: universal characterization of bacteria from domain to strain. Microbiology (Reading) 158:1005–1015. doi:10.1099/mic.0.055459-0 22282518 PMC3492749

[38] O’Leary NA , Wright MW , Brister JR , Ciufo S , Haddad D , McVeigh R , Rajput B , Robbertse B , Smith-White B , Ako-Adjei D , Astashyn A , Badretdin A , Bao Y , Blinkova O , Brover V , Chetvernin V , Choi J , Cox E , Ermolaeva O , Farrell CM , Goldfarb T , Gupta T , Haft D , Hatcher E , Hlavina W , Joardar VS , Kodali VK , Li W , Maglott D , Masterson P , McGarvey KM , Murphy MR , O’Neill K , Pujar S , Rangwala SH , Rausch D , Riddick LD , Schoch C , Shkeda A , Storz SS , Sun H , Thibaud-Nissen F , Tolstoy I , Tully RE , Vatsan AR , Wallin C , Webb D , Wu W , Landrum MJ , Kimchi A , Tatusova T , DiCuccio M , Kitts P , Murphy TD , Pruitt KD . 2016. Reference sequence (RefSeq) database at NCBI: current status, taxonomic expansion, and functional annotation. Nucleic Acids Res 44:D733–D745. doi:10.1093/nar/gkv1189 26553804 PMC4702849

[39] Wick RR , Judd LM , Gorrie CL , Holt KE . 2017. Unicycler: resolving bacterial genome assemblies from short and long sequencing reads. PLoS Comput Biol 13:e1005595. doi:10.1371/journal.pcbi.1005595 28594827 PMC5481147

[40] Gurevich A , Saveliev V , Vyahhi N , Tesler G . 2013. QUAST: quality assessment tool for genome assemblies. Bioinformatics 29:1072–1075. doi:10.1093/bioinformatics/btt086 23422339 PMC3624806

[41] Gilchrist CLM , Chooi Y-H . 2021. Clinker & Clustermap.Js: automatic generation of gene cluster comparison figures. Bioinformatics 37:2473–2475. doi:10.1093/bioinformatics/btab007 33459763

[42] Skinnider MA , Johnston CW , Gunabalasingam M , Merwin NJ , Kieliszek AM , MacLellan RJ , Li H , Ranieri MRM , Webster ALH , Cao MPT , Pfeifle A , Spencer N , To QH , Wallace DP , Dejong CA , Magarvey NA . 2020. Comprehensive prediction of secondary metabolite structure and biological activity from microbial genome sequences. Nat Commun 11:6058. doi:10.1038/s41467-020-19986-1 33247171 PMC7699628

[43] Carroll LM , Larralde M , Fleck JS , Ponnudurai R , Milanese A , Cappio E , Zeller G . 2021 Accurate de novo identification of biosynthetic gene clusters with GECCO . bioRxiv. doi:10.1101/2021.05.03.442509

[44] de Jong A , van Hijum SAFT , Bijlsma JJE , Kok J , Kuipers OP . 2006. BAGEL: a web-based bacteriocin genome mining tool. Nucleic Acids Res 34:W273–9. doi:10.1093/nar/gkl237 16845009 PMC1538908

[45] Zhao S , Guo Y , Sheng Q , Shyr Y . 2014. Heatmap3: an improved heatmap package with more powerful and convenient features. BMC Bioinformatics 15. doi:10.1186/1471-2105-15-S10-P16

[46] Gulick AM . 2017. Nonribosomal peptide synthetase biosynthetic clusters of ESKAPE pathogens. Nat Prod Rep 34:981–1009. doi:10.1039/c7np00029d 28642945 PMC5551671

[47] Zhao X , Li Z , Kuipers OP . 2020. Mimicry of a non-ribosomally produced antimicrobial, brevicidine, by ribosomal synthesis and post-translational modification. Cell Chem Biol 27:1262–1271. doi:10.1016/j.chembiol.2020.07.005 32707039

[48] Bushin LB , Covington BC , Rued BE , Federle MJ , Seyedsayamdost MR . 2020. Discovery and biosynthesis of streptosactin, a sactipeptide with an alternative topology encoded by commensal bacteria in the human microbiome. J Am Chem Soc 142:16265–16275. doi:10.1021/jacs.0c05546 32845143

[49] Shimamura H , Gouda H , Nagai K , Hirose T , Ichioka M , Furuya Y , Kobayashi Y , Hirono S , Sunazuka T , Omura S . 2009. Structure determination and total synthesis of bottromycin A2: a potent antibiotic against MRSA and VRE. Angew Chem Int Ed Engl 48:914–917. doi:10.1002/anie.200804138 19115340

[50] van Heel AJ , de Jong A , Song C , Viel JH , Kok J , Kuipers OP . 2018. BAGEL4: a user-friendly web server to thoroughly mine RiPPs and bacteriocins. Nucleic Acids Res 46:W278–W281. doi:10.1093/nar/gky383 29788290 PMC6030817

[51] Barreteau H , Tiouajni M , Graille M , Josseaume N , Bouhss A , Patin D , Blanot D , Fourgeaud M , Mainardi J-L , Arthur M , van Tilbeurgh H , Mengin-Lecreulx D , Touzé T . 2012. Functional and structural characterization of PaeM, a colicin M-like bacteriocin produced by Pseudomonas aeruginosa. J Biol Chem 287:37395–37405. doi:10.1074/jbc.M112.406439 22977250 PMC3481336

[52] Ghequire MGK , Öztürk B . 2018. A colicin M-type bacteriocin from Pseudomonas aeruginosa targeting the HxuC Heme receptor requires a novel immunity partner. Appl Environ Microbiol 84:1–11. doi:10.1128/AEM.00716-18 PMC6121995 29980560

[53] Costa SS , Lago LAB , Silva A , Graças DA das , Lameira J , Baraúna RA . 2022. Diversity of bacteriocins in the microbiome of the Tucuruí hydroelectric power plant water reservoir and three-dimensional structure prediction of a zoocin. Genet Mol Biol 45:e20210204. doi:10.1590/1678-4685-GMB-2021-0204 35037933 PMC8762718

[54] Rooney WM , Chai R , Milner JJ , Walker D . 2020. Bacteriocins targeting gram-negative phytopathogenic bacteria: plantibiotics of the future. Front Microbiol 11:575981. doi:10.3389/fmicb.2020.575981 33042091 PMC7530242

[55] Yang SC , Lin CH , Sung CT , Fang JY . 2014. Antibacterial activities of bacteriocins: application in foods and pharmaceuticals. Front Microbiol 5:683. doi:10.3389/fmicb.2014.00683 25544112 PMC4257098

[56] Denkovskienė E , Paškevičius Š , Misiūnas A , Stočkūnaitė B , Starkevič U , Vitkauskienė A , Hahn-Löbmann S , Schulz S , Giritch A , Gleba Y , Ražanskienė A . 2019. Broad and efficient control of Klebsiella pathogens by peptidoglycan-degrading and pore-forming bacteriocins klebicins. Sci Rep 9:15422. doi:10.1038/s41598-019-51969-1 31659220 PMC6817936

[57] Petrillo C , Castaldi S , Lanzilli M , Selci M , Cordone A , Giovannelli D , Isticato R . 2021. Genomic and physiological characterization of bacilli isolated from salt-pans with plant growth promoting features. Front Microbiol 12:715678. doi:10.3389/fmicb.2021.715678 34589073 PMC8475271

[58] Chukamnerd A , Pomwised R , Jeenkeawpiam K , Sakunrang C , Chusri S , Surachat K . 2022. Genomic insights into blaNDM-carrying carbapenem-resistant Klebsiella pneumoniae clinical isolates from a university hospital in Thailand. Microbiol Res 263:127136. doi:10.1016/j.micres.2022.127136 35870342

[59] Rebuffat S . 2011. Bacteriocins from gram-negative bacteria: a classification?, p 55–72. In Prokaryotic antimicrobial peptides. doi:10.1007/978-1-4419-7692-5

[60] Lu S , Wang J , Chitsaz F , Derbyshire MK , Geer RC , Gonzales NR , Gwadz M , Hurwitz DI , Marchler GH , Song JS , Thanki N , Yamashita RA , Yang M , Zhang D , Zheng C , Lanczycki CJ , Marchler-Bauer A . 2020. CDD/SPARCLE: the conserved domain database in 2020. Nucleic Acids Res 48:D265–D268. doi:10.1093/nar/gkz991 31777944 PMC6943070

[61] Eyles TH , Vior NM , Lacret R , Truman AW . 2021. Understanding thioamitide biosynthesis using pathway engineering and untargeted metabolomics. Chem Sci 12:7138–7150. doi:10.1039/d0sc06835g 34123341 PMC8153245

[62] Engelbrecht A , Saad H , Gross H , Kaysser L . 2021. Natural products from nocardia and their role in pathogenicity. Microb Physiol 31:217–232. doi:10.1159/000516864 34139700

[63] Sheldon JR , Skaar EP , Weiss DS . 2020. Acinetobacter baumannii can use multiple siderophores for iron acquisition, but only acinetobactin is required for virulence. PLoS Pathog. 16:e1008995. doi:10.1371/journal.ppat.1008995 33075115 PMC7595644

[64] Lee WC , Choi S , Jang A , Yeon J , Hwang E , Kim Y . 2021. Structural basis of the complementary activity of two ketosynthases in aryl polyene biosynthesis. Sci Rep 11:16340. doi:10.1038/s41598-021-95890-y 34381152 PMC8358021

[65] Metelev M , Arseniev A , Bushin LB , Kuznedelov K , Artamonova TO , Kondratenko R , Khodorkovskii M , Seyedsayamdost MR , Severinov K . 2017. Acinetodin and klebsidin, RNA polymerase targeting lasso peptides produced by human isolates of Acinetobacter gyllenbergii and Klebsiella pneumoniae. ACS Chem Biol 12:814–824. doi:10.1021/acschembio.6b01154 28106375

[66] Bailey DC , Alexander E , Rice MR , Drake EJ , Mydy LS , Aldrich CC , Gulick AM . 2018. Structural and functional delineation of aerobactin biosynthesis in hypervirulent Klebsiella pneumoniae. J Biol Chem 293:7841–7852. doi:10.1074/jbc.RA118.002798 29618511 PMC5961048

